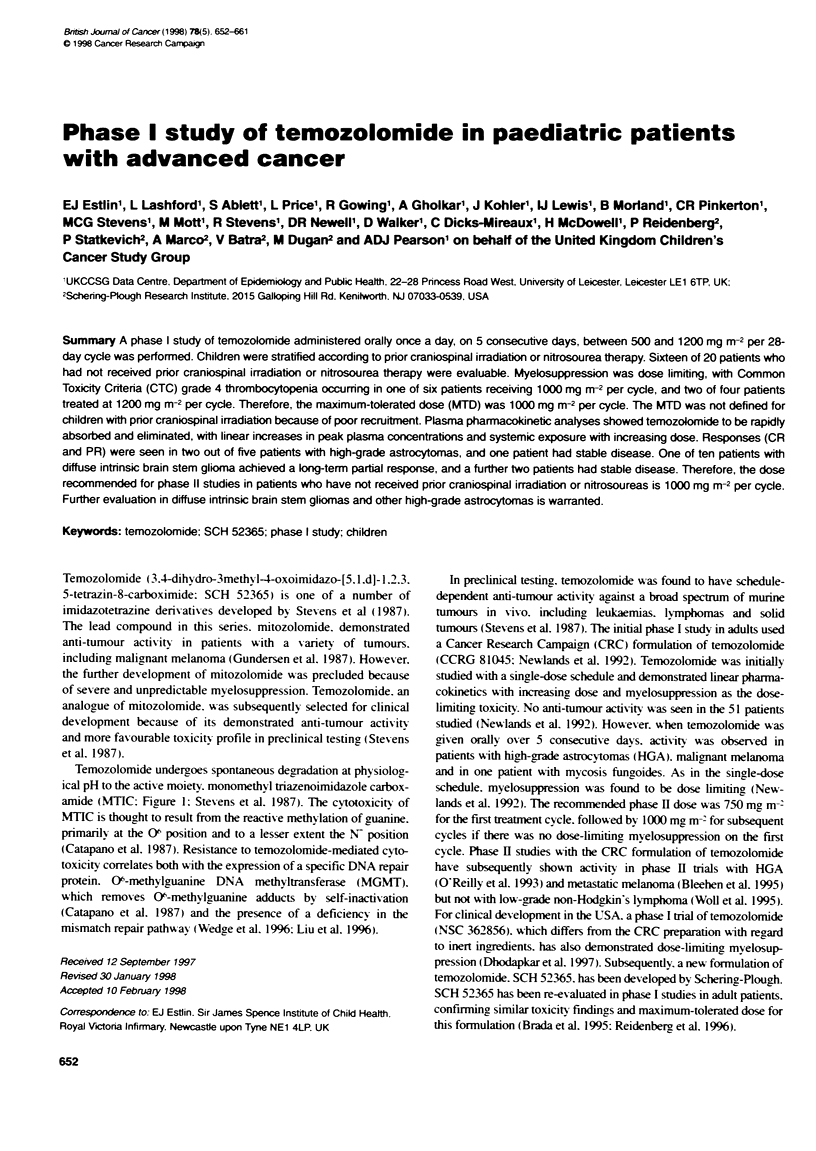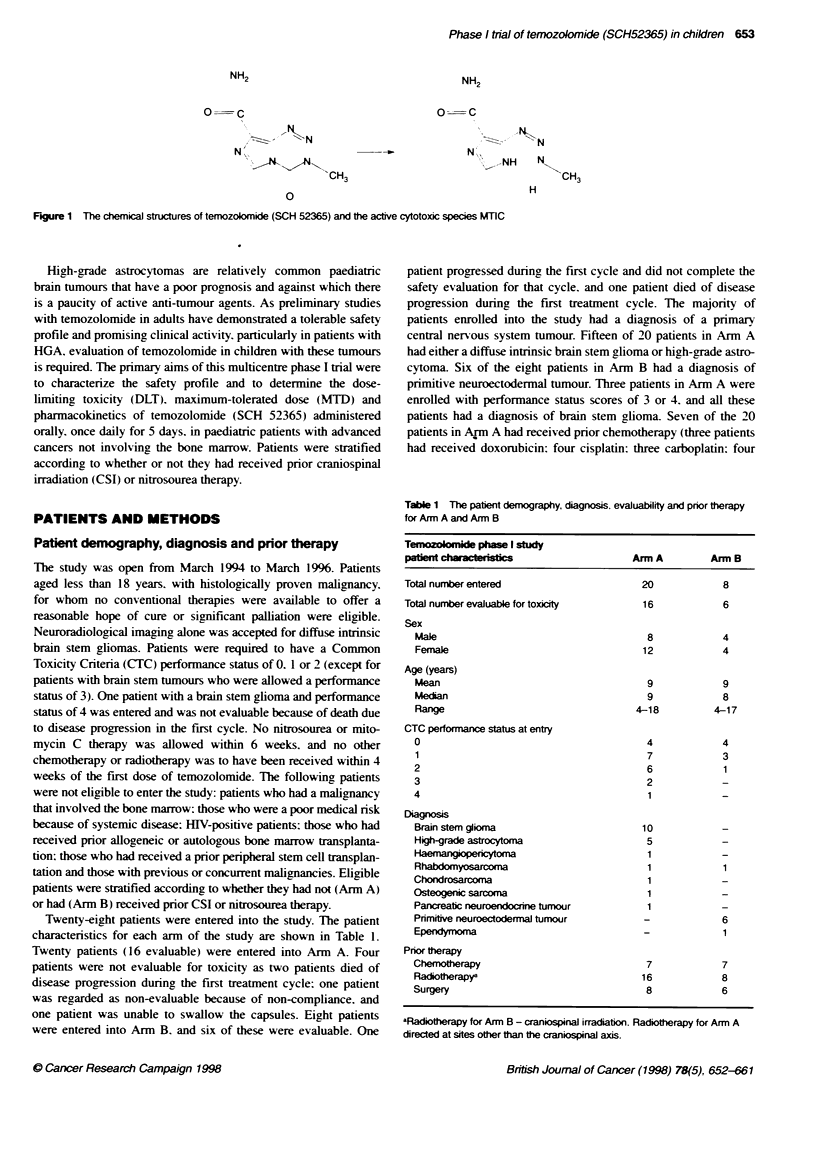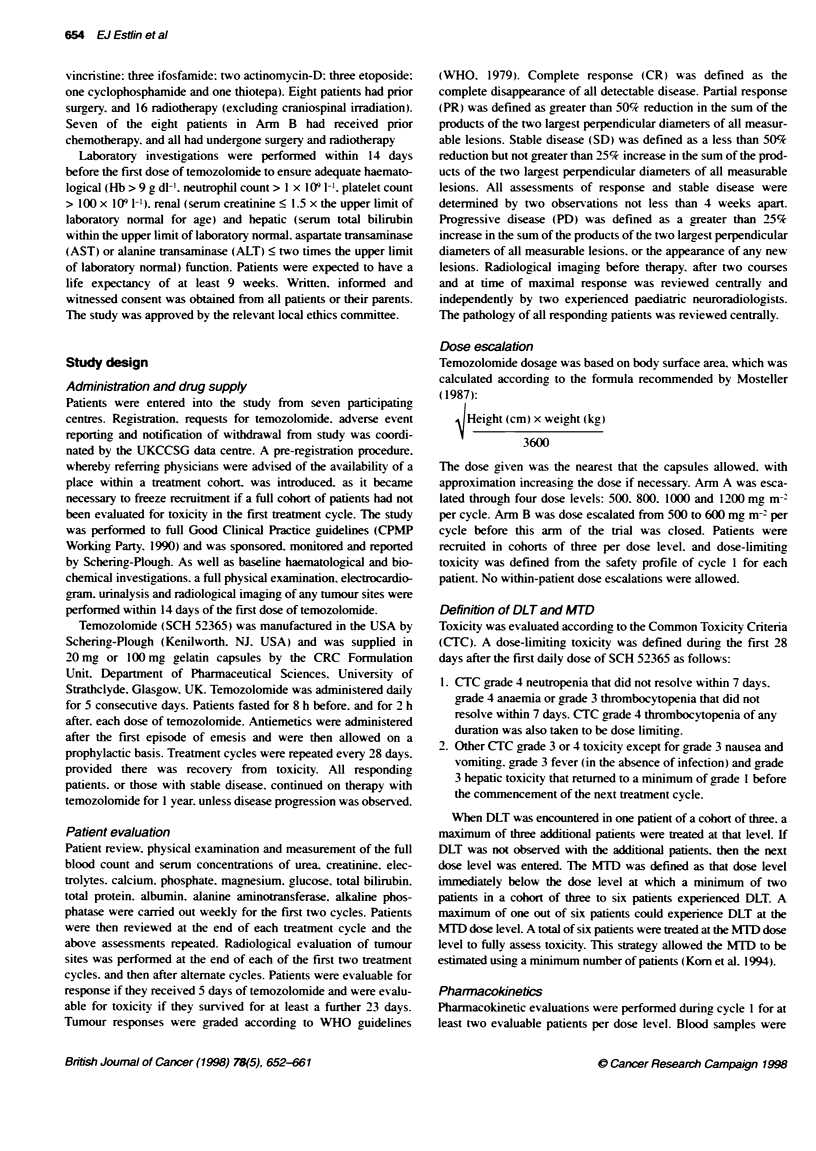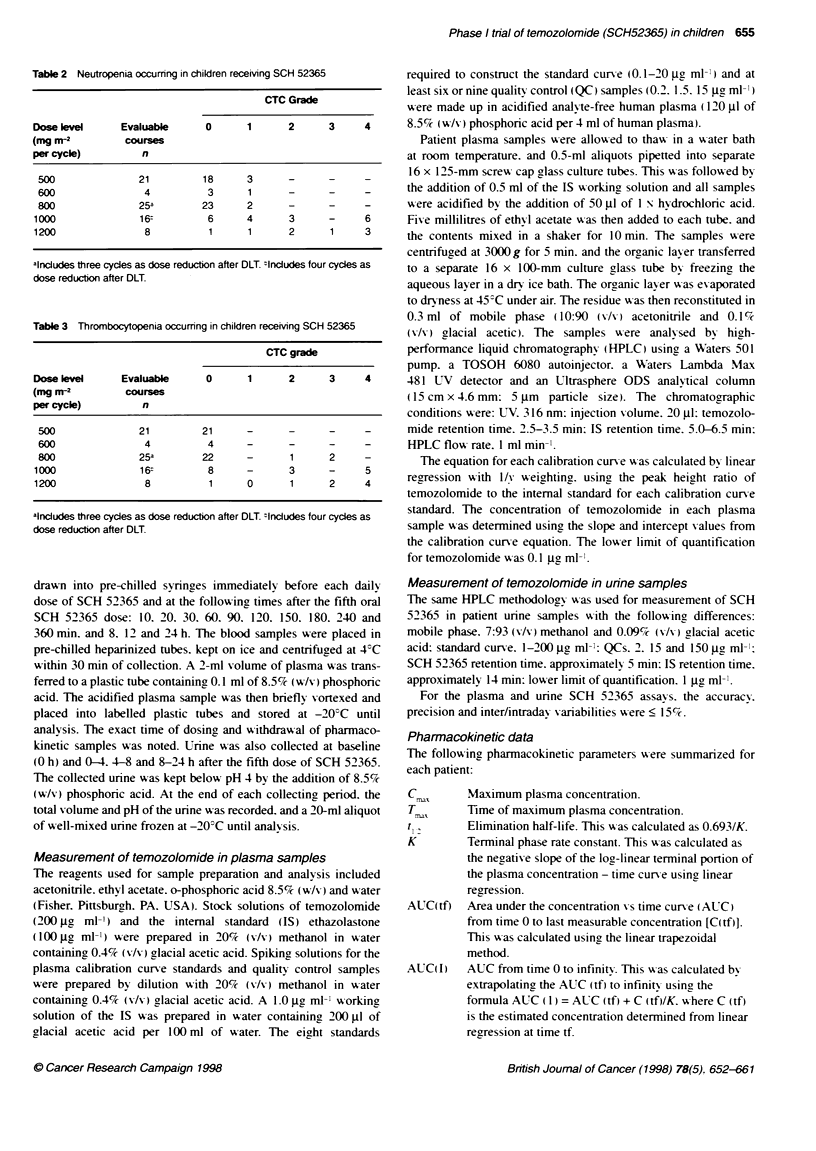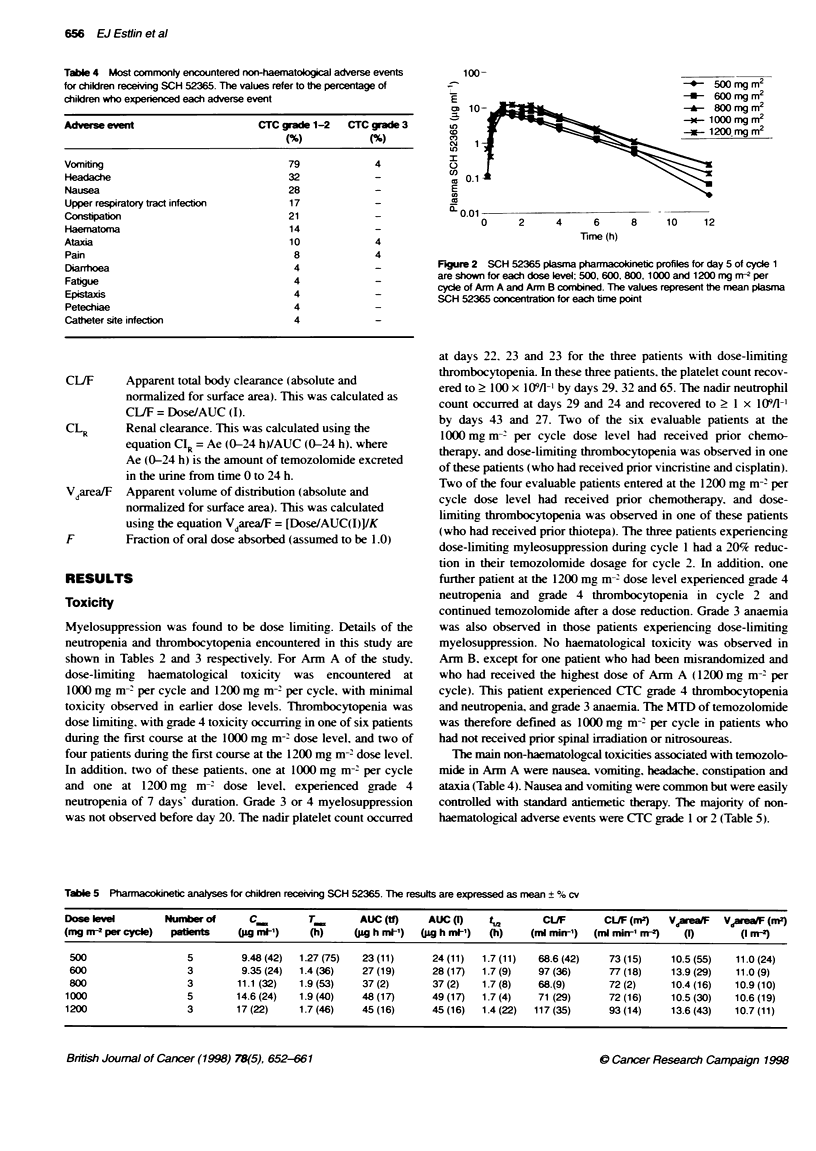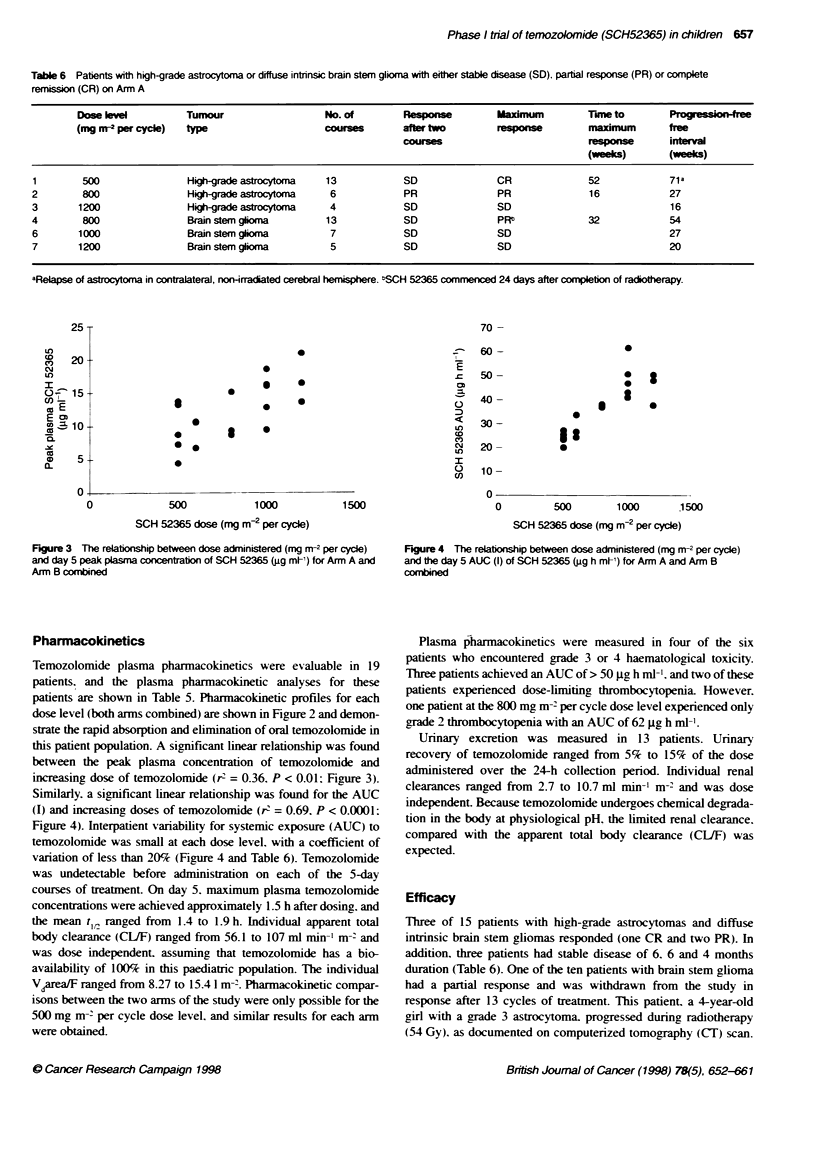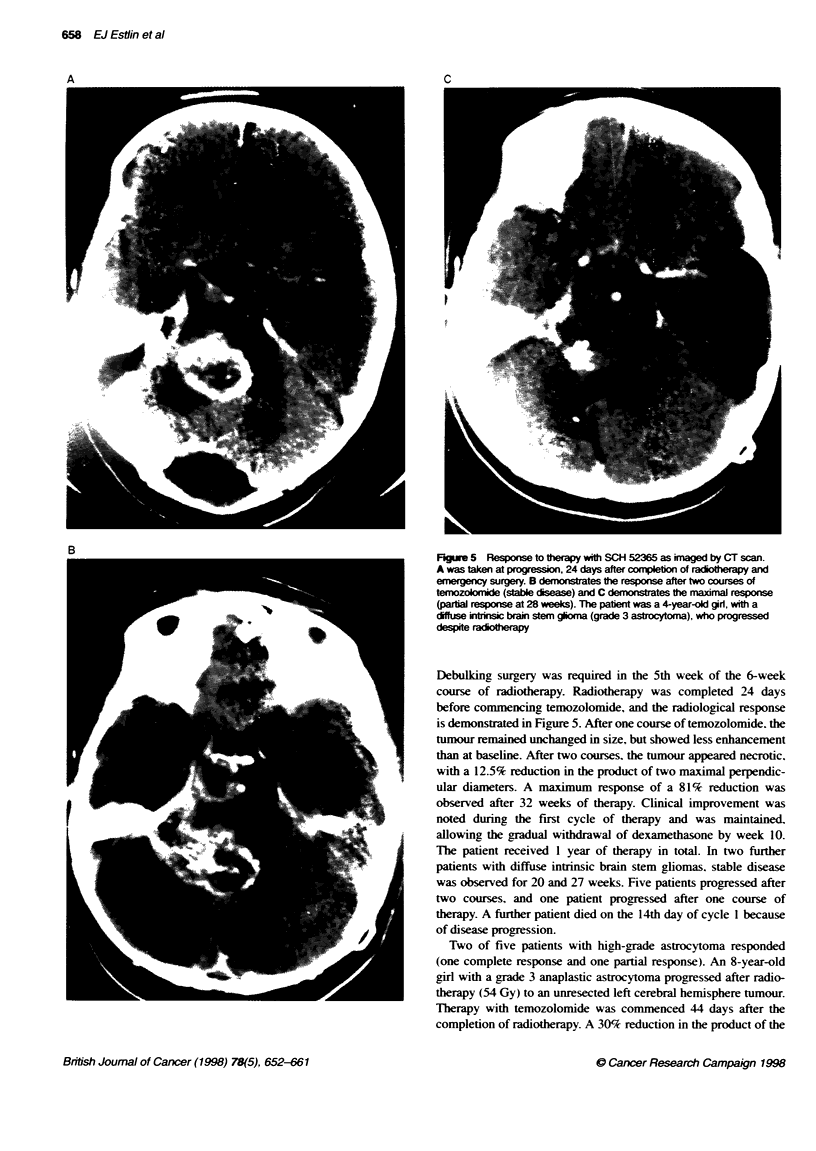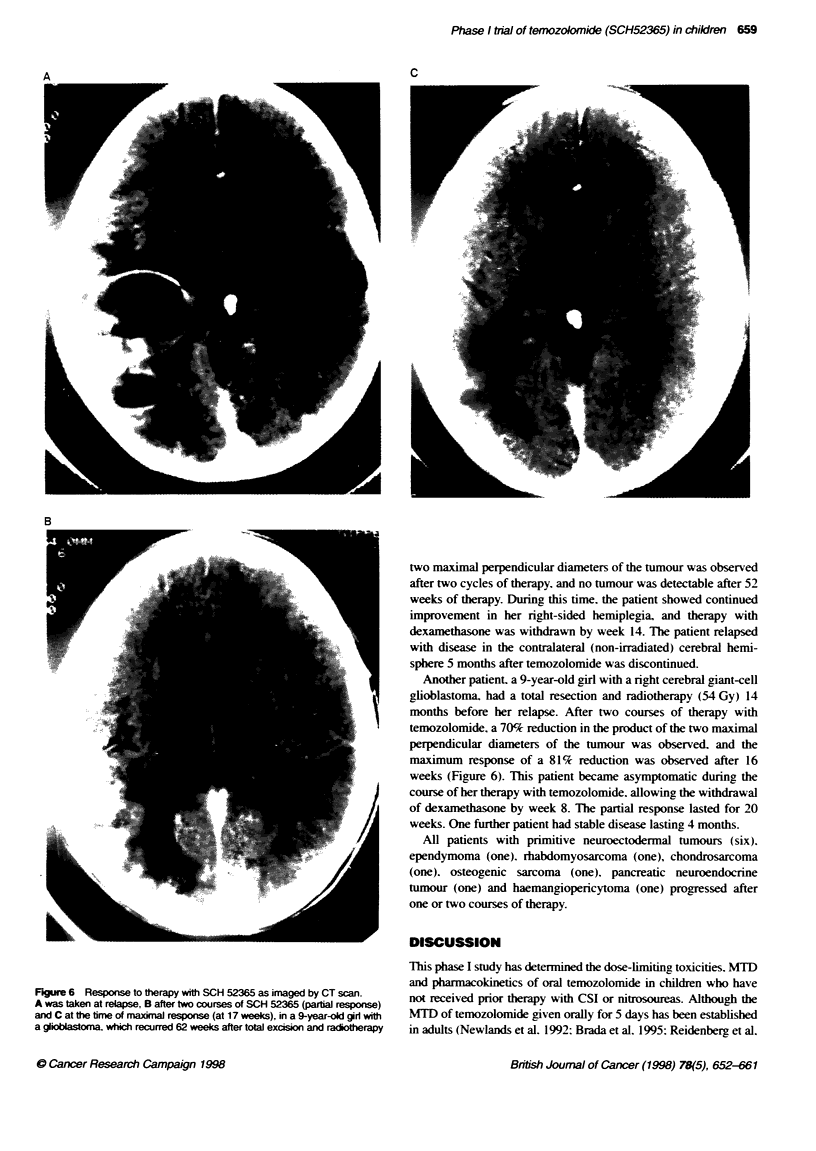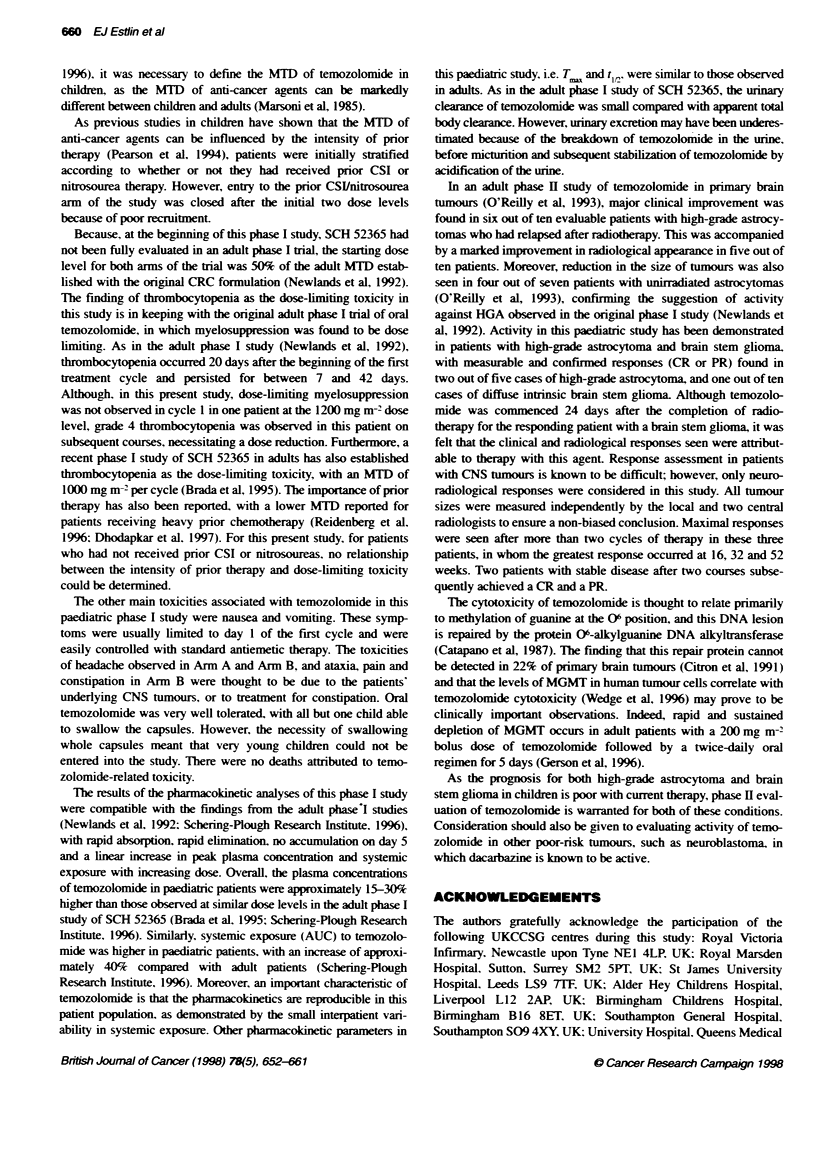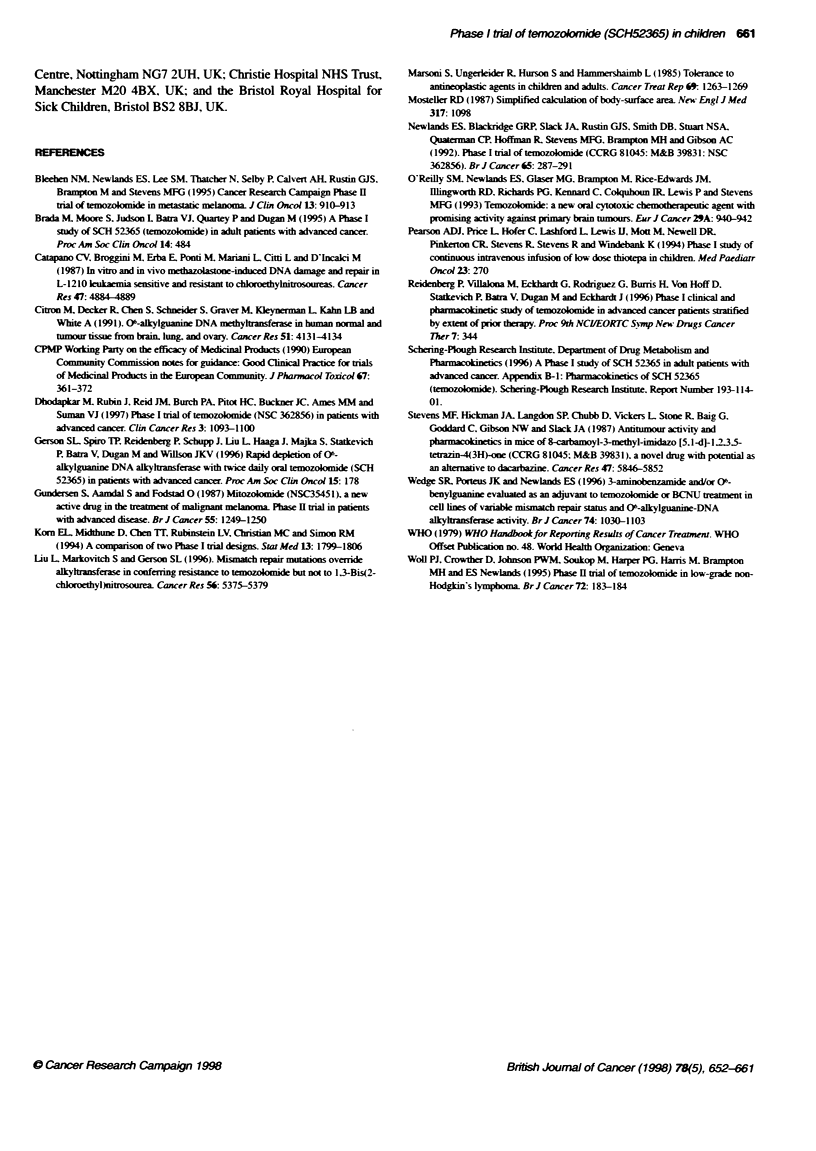# Phase I study of temozolomide in paediatric patients with advanced cancer. United Kingdom Children's Cancer Study Group.

**DOI:** 10.1038/bjc.1998.555

**Published:** 1998-09

**Authors:** E. J. Estlin, L. Lashford, S. Ablett, L. Price, R. Gowing, A. Gholkar, J. Kohler, I. J. Lewis, B. Morland, C. R. Pinkerton, M. C. Stevens, M. Mott, R. Stevens, D. R. Newell, D. Walker, C. Dicks-Mireaux, H. McDowell, P. Reidenberg, P. Statkevich, A. Marco, V. Batra, M. Dugan, A. D. Pearson

**Affiliations:** UKCCSG Data Centre, Department of Epidemiology and Public Health, University of Leicester, UK.

## Abstract

**Images:**


British Journal of Cancer (1998) 78(5), 652–661
© 1998 Cancer Research Campaign

### Phase I study of temozolomide in paediatric patients with advanced cancer

**EJ Estlin[1], L Lashford[1], S Ablett[1], L Price[1], R Gowing[1], A Gholkar[1], J Kohler[1], IJ Lewis[1], B Morland[1], CR Pinkerton[1], MCG Stevens[1], M Mott[1], R Stevens[1], DR Newell[1], D Walker[1], C Dicks-Mireaux[1], H McDowell[1], P Reidenberg[2], P Statkevich[2], A Marco[2], V Batra[2], M Dugan[2] and ADJ Pearson[1] on behalf of the United Kingdom Children's Cancer Study Group**

[1]UKCCSG Data Centre, Department of Epidemiology and Public Health, 22–28 Princess Road West, University of Leicester, Leicester LE1 6TP, UK; [2]Schering-Plough Research Institute, 2015 Galloping Hill Rd, Kenilworth, NJ 07033-0539, USA

**Summary** A phase I study of temozolomide administered orally once a day, on 5 consecutive days, between 500 and 1200 mg $m^{-2}$ per 28-day cycle was performed. Children were stratified according to prior craniospinal irradiation or nitrosourea therapy. Sixteen of 20 patients who had not received prior craniospinal irradiation or nitrosourea therapy were evaluable. Myelosuppression was dose limiting, with Common Toxicity Criteria (CTC) grade 4 thrombocytopenia occurring in one of six patients receiving 1000 mg $m^{-2}$ per cycle, and two of four patients treated at 1200 mg $m^{-2}$ per cycle. Therefore, the maximum-tolerated dose (MTD) was 1000 mg $m^{-2}$ per cycle. The MTD was not defined for children with prior craniospinal irradiation because of poor recruitment. Plasma pharmacokinetic analyses showed temozolomide to be rapidly absorbed and eliminated, with linear increases in peak plasma concentrations and systemic exposure with increasing dose. Responses (CR and PR) were seen in two out of five patients with high-grade astrocytomas, and one patient had stable disease. One of ten patients with diffuse intrinsic brain stem glioma achieved a long-term partial response, and a further two patients had stable disease. Therefore, the dose recommended for phase II studies in patients who have not received prior craniospinal irradiation or nitrosoureas is 1000 mg $m^{-2}$ per cycle. Further evaluation in diffuse intrinsic brain stem gliomas and other high-grade astrocytomas is warranted.

**Keywords:** temozolomide; SCH 52365; phase I study; children

Temozolomide (3,4-dihydro-3methyl-4-oxoimidazo-[5,1,d]-1,2,3, 5-tetrazin-8-carboximide; SCH 52365) is one of a number of imidazotetrazine derivatives developed by Stevens et al (1987). The lead compound in this series, mitozolomide, demonstrated anti-tumour activity in patients with a variety of tumours, including malignant melanoma (Gundersen et al, 1987). However, the further development of mitozolomide was precluded because of severe and unpredictable myelosuppression. Temozolomide, an analogue of mitozolomide, was subsequently selected for clinical development because of its demonstrated anti-tumour activity and more favourable toxicity profile in preclinical testing (Stevens et al, 1987).

Temozolomide undergoes spontaneous degradation at physiological pH to the active moiety, monomethyl triazenoimidazole carboxamide (MTIC; Figure 1; Stevens et al, 1987). The cytotoxicity of MTIC is thought to result from the reactive methylation of guanine, primarily at the $O^6$ position and to a lesser extent the $N^7$ position (Catapano et al, 1987). Resistance to temozolomide-mediated cytotoxicity correlates both with the expression of a specific DNA repair protein, $O^6$-methylguanine DNA methyltransferase (MGMT), which removes $O^6$-methylguanine adducts by self-inactivation (Catapano et al, 1987) and the presence of a deficiency in the mismatch repair pathway (Wedge et al, 1996; Liu et al, 1996).

*Received 12 September 1997*
*Revised 30 January 1998*
*Accepted 10 February 1998*

*Correspondence to:* EJ Estlin, Sir James Spence Institute of Child Health, Royal Victoria Infirmary, Newcastle upon Tyne NE1 4LP, UK

In preclinical testing, temozolomide was found to have schedule-dependent anti-tumour activity against a broad spectrum of murine tumours in vivo, including leukaemias, lymphomas and solid tumours (Stevens et al, 1987). The initial phase I study in adults used a Cancer Research Campaign (CRC) formulation of temozolomide (CCRG 81045; Newlands et al, 1992). Temozolomide was initially studied with a single-dose schedule and demonstrated linear pharmacokinetics with increasing dose and myelosuppression as the dose-limiting toxicity. No anti-tumour activity was seen in the 51 patients studied (Newlands et al, 1992). However, when temozolomide was given orally over 5 consecutive days, activity was observed in patients with high-grade astrocytomas (HGA), malignant melanoma and in one patient with mycosis fungoides. As in the single-dose schedule, myelosuppression was found to be dose limiting (Newlands et al, 1992). The recommended phase II dose was 750 mg $m^{-2}$ for the first treatment cycle, followed by 1000 mg $m^{-2}$ for subsequent cycles if there was no dose-limiting myelosuppression on the first cycle. Phase II studies with the CRC formulation of temozolomide have subsequently shown activity in phase II trials with HGA (O'Reilly et al, 1993) and metastatic melanoma (Bleehen et al, 1995) but not with low-grade non-Hodgkin's lymphoma (Woll et al, 1995). For clinical development in the USA, a phase I trial of temozolomide (NSC 362856), which differs from the CRC preparation with regard to inert ingredients, has also demonstrated dose-limiting myelosuppression (Dhodapkar et al, 1997). Subsequently, a new formulation of temozolomide, SCH 52365, has been developed by Schering-Plough. SCH 52365 has been re-evaluated in phase I studies in adult patients, confirming similar toxicity findings and maximum-tolerated dose for this formulation (Brada et al, 1995; Reidenberg et al, 1996).

**Figure 1** The chemical structures of temozolomide (SCH 52365) and the active cytotoxic species MTIC

High-grade astrocytomas are relatively common paediatric brain tumours that have a poor prognosis and against which there is a paucity of active anti-tumour agents. As preliminary studies with temozolomide in adults have demonstrated a tolerable safety profile and promising clinical activity, particularly in patients with HGA, evaluation of temozolomide in children with these tumours is required. The primary aims of this multicentre phase I trial were to characterize the safety profile and to determine the dose-limiting toxicity (DLT), maximum-tolerated dose (MTD) and pharmacokinetics of temozolomide (SCH 52365) administered orally, once daily for 5 days, in paediatric patients with advanced cancers not involving the bone marrow. Patients were stratified according to whether or not they had received prior craniospinal irradiation (CSI) or nitrosourea therapy.

### PATIENTS AND METHODS

### Patient demography, diagnosis and prior therapy

The study was open from March 1994 to March 1996. Patients aged less than 18 years, with histologically proven malignancy, for whom no conventional therapies were available to offer a reasonable hope of cure or significant palliation were eligible. Neuroradiological imaging alone was accepted for diffuse intrinsic brain stem gliomas. Patients were required to have a Common Toxicity Criteria (CTC) performance status of 0, 1 or 2 (except for patients with brain stem tumours who were allowed a performance status of 3). One patient with a brain stem glioma and performance status of 4 was entered and was not evaluable because of death due to disease progression in the first cycle. No nitrosourea or mitomycin C therapy was allowed within 6 weeks, and no other chemotherapy or radiotherapy was to have been received within 4 weeks of the first dose of temozolomide. The following patients were not eligible to enter the study: patients who had a malignancy that involved the bone marrow; those who were a poor medical risk because of systemic disease; HIV-positive patients; those who had received prior allogeneic or autologous bone marrow transplantation; those who had received a prior peripheral stem cell transplantation and those with previous or concurrent malignancies. Eligible patients were stratified according to whether they had not (Arm A) or had (Arm B) received prior CSI or nitrosourea therapy.

Twenty-eight patients were entered into the study. The patient characteristics for each arm of the study are shown in Table 1. Twenty patients (16 evaluable) were entered into Arm A. Four patients were not evaluable for toxicity as two patients died of disease progression during the first treatment cycle; one patient was regarded as non-evaluable because of non-compliance, and one patient was unable to swallow the capsules. Eight patients were entered into Arm B, and six of these were evaluable. One patient progressed during the first cycle and did not complete the safety evaluation for that cycle, and one patient died of disease progression during the first treatment cycle. The majority of patients enrolled into the study had a diagnosis of a primary central nervous system tumour. Fifteen of 20 patients in Arm A had either a diffuse intrinsic brain stem glioma or high-grade astrocytoma. Six of the eight patients in Arm B had a diagnosis of primitive neuroectodermal tumour. Three patients in Arm A were enrolled with performance status scores of 3 or 4, and all these patients had a diagnosis of brain stem glioma. Seven of the 20 patients in Arm A had received prior chemotherapy (three patients had received doxorubicin; four cisplatin; three carboplatin; four

**Table 1** The patient demography, diagnosis, evaluability and prior therapy for Arm A and Arm B

| Temozolomide phase I study patient characteristics | Arm A | Arm B |
|---|---|---|
| Total number entered | 20 | 8 |
| Total number evaluable for toxicity | 16 | 6 |
| Sex | | |
| Male | 8 | 4 |
| Female | 12 | 4 |
| Age (years) | | |
| Mean | 9 | 9 |
| Median | 9 | 8 |
| Range | 4–18 | 4–17 |
| CTC performance status at entry | | |
| 0 | 4 | 4 |
| 1 | 7 | 3 |
| 2 | 6 | 1 |
| 3 | 2 | – |
| 4 | 1 | – |
| Diagnosis | | |
| Brain stem glioma | 10 | – |
| High-grade astrocytoma | 5 | – |
| Haemangiopericytoma | 1 | – |
| Rhabdomyosarcoma | 1 | 1 |
| Chondrosarcoma | 1 | – |
| Osteogenic sarcoma | 1 | – |
| Pancreatic neuroendocrine tumour | 1 | – |
| Primitive neuroectodermal tumour | – | 6 |
| Ependymoma | – | 1 |
| Prior therapy | | |
| Chemotherapy | 7 | 7 |
| Radiotherapy[a] | 16 | 8 |
| Surgery | 8 | 6 |

[a]Radiotherapy for Arm B – craniospinal irradiation. Radiotherapy for Arm A directed at sites other than the craniospinal axis.

© Cancer Research Campaign 1998

vincristine: three ifosfamide: two actinomycin-D: three etoposide: one cyclophosphamide and one thiotepa). Eight patients had prior surgery. and 16 radiotherapy (excluding craniospinal irradiation). Seven of the eight patients in Arm B had received prior chemotherapy. and all had undergone surgery and radiotherapy

Laboratory investigations were performed within 14 days before the first dose of temozolomide to ensure adequate haematological (Hb > 9 g $dl^{-1}$. neutrophil count > 1 × $10^9$ $l^{-1}$. platelet count > 100 × $10^9$ $l^{-1}$). renal (serum creatinine ≤ 1.5 × the upper limit of laboratory normal for age) and hepatic (serum total bilirubin within the upper limit of laboratory normal. aspartate transaminase (AST) or alanine transaminase (ALT) ≤ two times the upper limit of laboratory normal) function. Patients were expected to have a life expectancy of at least 9 weeks. Written. informed and witnessed consent was obtained from all patients or their parents. The study was approved by the relevant local ethics committee.

### Study design

### Administration and drug supply

Patients were entered into the study from seven participating centres. Registration. requests for temozolomide. adverse event reporting and notification of withdrawal from study was coordinated by the UKCCSG data centre. A pre-registration procedure. whereby referring physicians were advised of the availability of a place within a treatment cohort. was introduced. as it became necessary to freeze recruitment if a full cohort of patients had not been evaluated for toxicity in the first treatment cycle. The study was performed to full Good Clinical Practice guidelines (CPMP Working Party. 1990) and was sponsored. monitored and reported by Schering-Plough. As well as baseline haematological and biochemical investigations. a full physical examination. electrocardiogram. urinalysis and radiological imaging of any tumour sites were performed within 14 days of the first dose of temozolomide.

Temozolomide (SCH 52365) was manufactured in the USA by Schering-Plough (Kenilworth. NJ. USA) and was supplied in 20 mg or 100 mg gelatin capsules by the CRC Formulation Unit. Department of Pharmaceutical Sciences. University of Strathclyde. Glasgow. UK. Temozolomide was administered daily for 5 consecutive days. Patients fasted for 8 h before. and for 2 h after. each dose of temozolomide. Antiemetics were administered after the first episode of emesis and were then allowed on a prophylactic basis. Treatment cycles were repeated every 28 days. provided there was recovery from toxicity. All responding patients. or those with stable disease. continued on therapy with temozolomide for 1 year. unless disease progression was observed.

### Patient evaluation

Patient review. physical examination and measurement of the full blood count and serum concentrations of urea. creatinine. electrolytes. calcium. phosphate. magnesium. glucose. total bilirubin. total protein. albumin. alanine aminotransferase. alkaline phosphatase were carried out weekly for the first two cycles. Patients were then reviewed at the end of each treatment cycle and the above assessments repeated. Radiological evaluation of tumour sites was performed at the end of each of the first two treatment cycles. and then after alternate cycles. Patients were evaluable for response if they received 5 days of temozolomide and were evaluable for toxicity if they survived for at least a further 23 days. Tumour responses were graded according to WHO guidelines (WHO. 1979). Complete response (CR) was defined as the complete disappearance of all detectable disease. Partial response (PR) was defined as greater than 50% reduction in the sum of the products of the two largest perpendicular diameters of all measurable lesions. Stable disease (SD) was defined as a less than 50% reduction but not greater than 25% increase in the sum of the products of the two largest perpendicular diameters of all measurable lesions. All assessments of response and stable disease were determined by two observations not less than 4 weeks apart. Progressive disease (PD) was defined as a greater than 25% increase in the sum of the products of the two largest perpendicular diameters of all measurable lesions. or the appearance of any new lesions. Radiological imaging before therapy. after two courses and at time of maximal response was reviewed centrally and independently by two experienced paediatric neuroradiologists. The pathology of all responding patients was reviewed centrally.

### Dose escalation

Temozolomide dosage was based on body surface area. which was calculated according to the formula recommended by Mosteller (1987):

$$\sqrt{\frac{\text{Height (cm)} \times \text{weight (kg)}}{3600}}$$

The dose given was the nearest that the capsules allowed. with approximation increasing the dose if necessary. Arm A was escalated through four dose levels: 500. 800. 1000 and 1200 mg $m^{-2}$ per cycle. Arm B was dose escalated from 500 to 600 mg $m^{-2}$ per cycle before this arm of the trial was closed. Patients were recruited in cohorts of three per dose level. and dose-limiting toxicity was defined from the safety profile of cycle 1 for each patient. No within-patient dose escalations were allowed.

### Definition of DLT and MTD

Toxicity was evaluated according to the Common Toxicity Criteria (CTC). A dose-limiting toxicity was defined during the first 28 days after the first daily dose of SCH 52365 as follows:

1. CTC grade 4 neutropenia that did not resolve within 7 days. grade 4 anaemia or grade 3 thrombocytopenia that did not resolve within 7 days. CTC grade 4 thrombocytopenia of any duration was also taken to be dose limiting.
2. Other CTC grade 3 or 4 toxicity except for grade 3 nausea and vomiting. grade 3 fever (in the absence of infection) and grade 3 hepatic toxicity that returned to a minimum of grade 1 before the commencement of the next treatment cycle.

When DLT was encountered in one patient of a cohort of three. a maximum of three additional patients were treated at that level. If DLT was not observed with the additional patients. then the next dose level was entered. The MTD was defined as that dose level immediately below the dose level at which a minimum of two patients in a cohort of three to six patients experienced DLT. A maximum of one out of six patients could experience DLT at the MTD dose level. A total of six patients were treated at the MTD dose level to fully assess toxicity. This strategy allowed the MTD to be estimated using a minimum number of patients (Korn et al. 1994).

### Pharmacokinetics

Pharmacokinetic evaluations were performed during cycle 1 for at least two evaluable patients per dose level. Blood samples were

 © Cancer Research Campaign 1998

**Table 2** Neutropenia occurring in children receiving SCH 52365

| **Dose level (mg m$^{-2}$ per cycle)** | **Evaluable courses *n*** | **CTC Grade** | | | | |
|---|---|---|---|---|---|---|
| | | **0** | **1** | **2** | **3** | **4** |
| 500 | 21 | 18 | 3 | – | – | – |
| 600 | 4 | 3 | 1 | – | – | – |
| 800 | 25[a] | 23 | 2 | – | – | – |
| 1000 | 16[b] | 6 | 4 | 3 | – | 6 |
| 1200 | 8 | 1 | 1 | 2 | 1 | 3 |

[a]Includes three cycles as dose reduction after DLT. [b]Includes four cycles as dose reduction after DLT.

**Table 3** Thrombocytopenia occurring in children receiving SCH 52365

| **Dose level (mg m$^{-2}$ per cycle)** | **Evaluable courses *n*** | **CTC grade** | | | | |
|---|---|---|---|---|---|---|
| | | **0** | **1** | **2** | **3** | **4** |
| 500 | 21 | 21 | – | – | – | – |
| 600 | 4 | 4 | – | – | – | – |
| 800 | 25[a] | 22 | – | 1 | 2 | – |
| 1000 | 16[b] | 8 | – | 3 | – | 5 |
| 1200 | 8 | 1 | 0 | 1 | 2 | 4 |

[a]Includes three cycles as dose reduction after DLT. [b]Includes four cycles as dose reduction after DLT.

drawn into pre-chilled syringes immediately before each daily dose of SCH 52365 and at the following times after the fifth oral SCH 52365 dose: 10, 20, 30, 60, 90, 120, 150, 180, 240 and 360 min, and 8, 12 and 24 h. The blood samples were placed in pre-chilled heparinized tubes, kept on ice and centrifuged at 4°C within 30 min of collection. A 2-ml volume of plasma was transferred to a plastic tube containing 0.1 ml of 8.5% (w/v) phosphoric acid. The acidified plasma sample was then briefly vortexed and placed into labelled plastic tubes and stored at –20°C until analysis. The exact time of dosing and withdrawal of pharmacokinetic samples was noted. Urine was also collected at baseline (0 h) and 0–4, 4–8 and 8–24 h after the fifth dose of SCH 52365. The collected urine was kept below pH 4 by the addition of 8.5% (w/v) phosphoric acid. At the end of each collecting period, the total volume and pH of the urine was recorded, and a 20-ml aliquot of well-mixed urine frozen at –20°C until analysis.

### *Measurement of temozolomide in plasma samples*

The reagents used for sample preparation and analysis included acetonitrile, ethyl acetate, o-phosphoric acid 8.5% (w/v) and water (Fisher, Pittsburgh, PA, USA). Stock solutions of temozolomide (200 µg ml$^{-1}$) and the internal standard (IS) ethazolastone (100 µg ml$^{-1}$) were prepared in 20% (v/v) methanol in water containing 0.4% (v/v) glacial acetic acid. Spiking solutions for the plasma calibration curve standards and quality control samples were prepared by dilution with 20% (v/v) methanol in water containing 0.4% (v/v) glacial acetic acid. A 1.0 µg ml$^{-1}$ working solution of the IS was prepared in water containing 200 µl of glacial acetic acid per 100 ml of water. The eight standards required to construct the standard curve (0.1–20 µg ml$^{-1}$) and at least six or nine quality control (QC) samples (0.2, 1.5, 15 µg ml$^{-1}$) were made up in acidified analyte-free human plasma (120 µl of 8.5% (w/v) phosphoric acid per 4 ml of human plasma).

Patient plasma samples were allowed to thaw in a water bath at room temperature, and 0.5-ml aliquots pipetted into separate 16 × 125-mm screw cap glass culture tubes. This was followed by the addition of 0.5 ml of the IS working solution and all samples were acidified by the addition of 50 µl of 1 N hydrochloric acid. Five millilitres of ethyl acetate was then added to each tube, and the contents mixed in a shaker for 10 min. The samples were centrifuged at 3000 *g* for 5 min, and the organic layer transferred to a separate 16 × 100-mm culture glass tube by freezing the aqueous layer in a dry ice bath. The organic layer was evaporated to dryness at 45°C under air. The residue was then reconstituted in 0.3 ml of mobile phase (10:90 (v/v) acetonitrile and 0.1% (v/v) glacial acetic). The samples were analysed by high-performance liquid chromatography (HPLC) using a Waters 501 pump, a TOSOH 6080 autoinjector, a Waters Lambda Max 481 UV detector and an Ultrasphere ODS analytical column (15 cm × 4.6 mm; 5 µm particle size). The chromatographic conditions were: UV, 316 nm; injection volume, 20 µl; temozolomide retention time, 2.5–3.5 min; IS retention time, 5.0–6.5 min; HPLC flow rate, 1 ml min$^{-1}$.

The equation for each calibration curve was calculated by linear regression with 1/y weighting, using the peak height ratio of temozolomide to the internal standard for each calibration curve standard. The concentration of temozolomide in each plasma sample was determined using the slope and intercept values from the calibration curve equation. The lower limit of quantification for temozolomide was 0.1 µg ml$^{-1}$.

### *Measurement of temozolomide in urine samples*

The same HPLC methodology was used for measurement of SCH 52365 in patient urine samples with the following differences: mobile phase, 7:93 (v/v) methanol and 0.09% (v/v) glacial acetic acid; standard curve, 1–200 µg ml$^{-1}$; QCs, 2, 15 and 150 µg ml$^{-1}$; SCH 52365 retention time, approximately 5 min; IS retention time, approximately 14 min; lower limit of quantification, 1 µg ml$^{-1}$.

For the plasma and urine SCH 52365 assays, the accuracy, precision and inter/intraday variabilities were ≤ 15%.

### *Pharmacokinetic data*

The following pharmacokinetic parameters were summarized for each patient:

| | |
|---|---|
| $C_{max}$ | Maximum plasma concentration. |
| $T_{max}$ | Time of maximum plasma concentration. |
| $t_{1/2}$ | Elimination half-life. This was calculated as 0.693/*K*. |
| *K* | Terminal phase rate constant. This was calculated as the negative slope of the log-linear terminal portion of the plasma concentration – time curve using linear regression. |
| AUC(tf) | Area under the concentration vs time curve (AUC) from time 0 to last measurable concentration [C(tf)]. This was calculated using the linear trapezoidal method. |
| AUC(I) | AUC from time 0 to infinity. This was calculated by extrapolating the AUC (tf) to infinity using the formula AUC (I) = AUC (tf) + C (tf)/*K*, where C (tf) is the estimated concentration determined from linear regression at time tf. |

Table 4 Most commonly encountered non-haematological adverse events for children receiving SCH 52365. The values refer to the percentage of children who experienced each adverse event

| Adverse event | CTC grade 1–2 (%) | CTC grade 3 (%) |
|---|---|---|
| Vomiting | 79 | 4 |
| Headache | 32 | – |
| Nausea | 28 | – |
| Upper respiratory tract infection | 17 | – |
| Constipation | 21 | – |
| Haematoma | 14 | – |
| Ataxia | 10 | 4 |
| Pain | 8 | 4 |
| Diarrhoea | 4 | – |
| Fatigue | 4 | – |
| Epistaxis | 4 | – |
| Petechiae | 4 | – |
| Catheter site infection | 4 | – |

CL/F — Apparent total body clearance (absolute and normalized for surface area). This was calculated as CL/F = Dose/AUC (I).

$CL_R$ — Renal clearance. This was calculated using the equation $CI_R$ = Ae (0–24 h)/AUC (0–24 h), where Ae (0–24 h) is the amount of temozolomide excreted in the urine from time 0 to 24 h.

$V_d$area/F — Apparent volume of distribution (absolute and normalized for surface area). This was calculated using the equation $V_d$area/F = [Dose/AUC(I)]/$K$

$F$ — Fraction of oral dose absorbed (assumed to be 1.0)

### RESULTS

### Toxicity

Myelosuppression was found to be dose limiting. Details of the neutropenia and thrombocytopenia encountered in this study are shown in Tables 2 and 3 respectively. For Arm A of the study, dose-limiting haematological toxicity was encountered at 1000 mg m$^{-2}$ per cycle and 1200 mg m$^{-2}$ per cycle, with minimal toxicity observed in earlier dose levels. Thrombocytopenia was dose limiting, with grade 4 toxicity occurring in one of six patients during the first course at the 1000 mg m$^{-2}$ dose level, and two of four patients during the first course at the 1200 mg m$^{-2}$ dose level. In addition, two of these patients, one at 1000 mg m$^{-2}$ per cycle and one at 1200 mg m$^{-2}$ dose level, experienced grade 4 neutropenia of 7 days' duration. Grade 3 or 4 myelosuppression was not observed before day 20. The nadir platelet count occurred

Figure 2 SCH 52365 plasma pharmacokinetic profiles for day 5 of cycle 1 are shown for each dose level; 500, 600, 800, 1000 and 1200 mg m$^{-2}$ per cycle of Arm A and Arm B combined. The values represent the mean plasma SCH 52365 concentration for each time point at days 22, 23 and 23 for the three patients with dose-limiting thrombocytopenia. In these three patients, the platelet count recovered to ≥ 100 × 10$^9$/l$^{-1}$ by days 29, 32 and 65. The nadir neutrophil count occurred at days 29 and 24 and recovered to ≥ 1 × 10$^9$/l$^{-1}$ by days 43 and 27. Two of the six evaluable patients at the 1000 mg m$^{-2}$ per cycle dose level had received prior chemotherapy, and dose-limiting thrombocytopenia was observed in one of these patients (who had received prior vincristine and cisplatin). Two of the four evaluable patients entered at the 1200 mg m$^{-2}$ per cycle dose level had received prior chemotherapy, and dose-limiting thrombocytopenia was observed in one of these patients (who had received prior thiotepa). The three patients experiencing dose-limiting myleosuppression during cycle 1 had a 20% reduction in their temozolomide dosage for cycle 2. In addition, one further patient at the 1200 mg m$^{-2}$ dose level experienced grade 4 neutropenia and grade 4 thrombocytopenia in cycle 2 and continued temozolomide after a dose reduction. Grade 3 anaemia was also observed in those patients experiencing dose-limiting myelosuppression. No haematological toxicity was observed in Arm B, except for one patient who had been misrandomized and who had received the highest dose of Arm A (1200 mg m$^{-2}$ per cycle). This patient experienced CTC grade 4 thrombocytopenia and neutropenia, and grade 3 anaemia. The MTD of temozolomide was therefore defined as 1000 mg m$^{-2}$ per cycle in patients who had not received prior spinal irradiation or nitrosoureas.

The main non-haematologcal toxicities associated with temozolomide in Arm A were nausea, vomiting, headache, constipation and ataxia (Table 4). Nausea and vomiting were common but were easily controlled with standard antiemetic therapy. The majority of non-haematological adverse events were CTC grade 1 or 2 (Table 5).

Table 5 Pharmacokinetic analyses for children receiving SCH 52365. The results are expressed as mean ± % cv

| Dose level (mg m$^{-2}$ per cycle) | Number of patients | $C_{max}$ (μg ml$^{-1}$) | $T_{max}$ (h) | AUC (tf) (μg h ml$^{-1}$) | AUC (I) (μg h ml$^{-1}$) | $t_{1/2}$ (h) | CL/F (ml min$^{-1}$) | CL/F (m$^2$) (ml min$^{-1}$ m$^{-2}$) | $V_d$area/F (l) | $V_d$area/F (m$^2$) (l m$^{-2}$) |
|---|---|---|---|---|---|---|---|---|---|---|
| 500 | 5 | 9.48 (42) | 1.27 (75) | 23 (11) | 24 (11) | 1.7 (11) | 68.6 (42) | 73 (15) | 10.5 (55) | 11.0 (24) |
| 600 | 3 | 9.35 (24) | 1.4 (36) | 27 (19) | 28 (17) | 1.7 (9) | 97 (36) | 77 (18) | 13.9 (29) | 11.0 (9) |
| 800 | 3 | 11.1 (32) | 1.9 (53) | 37 (2) | 37 (2) | 1.7 (8) | 68.(9) | 72 (2) | 10.4 (16) | 10.9 (10) |
| 1000 | 5 | 14.6 (24) | 1.9 (40) | 48 (17) | 49 (17) | 1.7 (4) | 71 (29) | 72 (16) | 10.5 (30) | 10.6 (19) |
| 1200 | 3 | 17 (22) | 1.7 (46) | 45 (16) | 45 (16) | 1.4 (22) | 117 (35) | 93 (14) | 13.6 (43) | 10.7 (11) |

© Cancer Research Campaign 1998

**Table 6** Patients with high-grade astrocytoma or diffuse intrinsic brain stem glioma with either stable disease (SD), partial response (PR) or complete remission (CR) on Arm A

| | Dose level (mg m$^{-2}$ per cycle) | Tumour type | No. of courses | Response after two courses | Maximum response | Time to maximum response (weeks) | Progression-free free interval (weeks) |
|---|---|---|---|---|---|---|---|
| 1 | 500 | High-grade astrocytoma | 13 | SD | CR | 52 | 71[a] |
| 2 | 800 | High-grade astrocytoma | 6 | PR | PR | 16 | 27 |
| 3 | 1200 | High-grade astrocytoma | 4 | SD | SD | | 16 |
| 4 | 800 | Brain stem glioma | 13 | SD | PR[b] | 32 | 54 |
| 6 | 1000 | Brain stem glioma | 7 | SD | SD | | 27 |
| 7 | 1200 | Brain stem glioma | 5 | SD | SD | | 20 |

[a]Relapse of astrocytoma in contralateral, non-irradiated cerebral hemisphere. [b]SCH 52365 commenced 24 days after completion of radiotherapy.

**Figure 3** The relationship between dose administered (mg m$^{-2}$ per cycle) and day 5 peak plasma concentration of SCH 52365 (μg ml$^{-1}$) for Arm A and Arm B combined

**Figure 4** The relationship between dose administered (mg m$^{-2}$ per cycle) and the day 5 AUC (I) of SCH 52365 (μg h ml$^{-1}$) for Arm A and Arm B combined

### Pharmacokinetics

Temozolomide plasma pharmacokinetics were evaluable in 19 patients, and the plasma pharmacokinetic analyses for these patients are shown in Table 5. Pharmacokinetic profiles for each dose level (both arms combined) are shown in Figure 2 and demonstrate the rapid absorption and elimination of oral temozolomide in this patient population. A significant linear relationship was found between the peak plasma concentration of temozolomide and increasing dose of temozolomide ($r^2 = 0.36$, $P < 0.01$; Figure 3). Similarly, a significant linear relationship was found for the AUC (I) and increasing doses of temozolomide ($r^2 = 0.69$, $P < 0.0001$; Figure 4). Interpatient variability for systemic exposure (AUC) to temozolomide was small at each dose level, with a coefficient of variation of less than 20% (Figure 4 and Table 6). Temozolomide was undetectable before administration on each of the 5-day courses of treatment. On day 5, maximum plasma temozolomide concentrations were achieved approximately 1.5 h after dosing, and the mean $t_{1/2}$ ranged from 1.4 to 1.9 h. Individual apparent total body clearance (CL/F) ranged from 56.1 to 107 ml min$^{-1}$ m$^{-2}$ and was dose independent, assuming that temozolomide has a bioavailability of 100% in this paediatric population. The individual $V_d$area/F ranged from 8.27 to 15.4 l m$^{-2}$. Pharmacokinetic comparisons between the two arms of the study were only possible for the 500 mg m$^{-2}$ per cycle dose level, and similar results for each arm were obtained.

Plasma pharmacokinetics were measured in four of the six patients who encountered grade 3 or 4 haematological toxicity. Three patients achieved an AUC of > 50 μg h ml$^{-1}$, and two of these patients experienced dose-limiting thrombocytopenia. However, one patient at the 800 mg m$^{-2}$ per cycle dose level experienced only grade 2 thrombocytopenia with an AUC of 62 μg h ml$^{-1}$.

Urinary excretion was measured in 13 patients. Urinary recovery of temozolomide ranged from 5% to 15% of the dose administered over the 24-h collection period. Individual renal clearances ranged from 2.7 to 10.7 ml min$^{-1}$ m$^{-2}$ and was dose independent. Because temozolomide undergoes chemical degradation in the body at physiological pH, the limited renal clearance, compared with the apparent total body clearance (CL/F) was expected.

### Efficacy

Three of 15 patients with high-grade astrocytomas and diffuse intrinsic brain stem gliomas responded (one CR and two PR). In addition, three patients had stable disease of 6, 6 and 4 months duration (Table 6). One of the ten patients with brain stem glioma had a partial response and was withdrawn from the study in response after 13 cycles of treatment. This patient, a 4-year-old girl with a grade 3 astrocytoma, progressed during radiotherapy (54 Gy), as documented on computerized tomography (CT) scan.

© Cancer Research Campaign 1998 

A

B

C

**Figure 5** Response to therapy with SCH 52365 as imaged by CT scan. **A** was taken at progression, 24 days after completion of radiotherapy and emergency surgery. **B** demonstrates the response after two courses of temozolomide (stable disease) and **C** demonstrates the maximal response (partial response at 28 weeks). The patient was a 4-year-old girl, with a diffuse intrinsic brain stem glioma (grade 3 astrocytoma), who progressed despite radiotherapy

Debulking surgery was required in the 5th week of the 6-week course of radiotherapy. Radiotherapy was completed 24 days before commencing temozolomide, and the radiological response is demonstrated in Figure 5. After one course of temozolomide, the tumour remained unchanged in size, but showed less enhancement than at baseline. After two courses, the tumour appeared necrotic, with a 12.5% reduction in the product of two maximal perpendicular diameters. A maximum response of a 81% reduction was observed after 32 weeks of therapy. Clinical improvement was noted during the first cycle of therapy and was maintained, allowing the gradual withdrawal of dexamethasone by week 10. The patient received 1 year of therapy in total. In two further patients with diffuse intrinsic brain stem gliomas, stable disease was observed for 20 and 27 weeks. Five patients progressed after two courses, and one patient progressed after one course of therapy. A further patient died on the 14th day of cycle 1 because of disease progression.

Two of five patients with high-grade astrocytoma responded (one complete response and one partial response). An 8-year-old girl with a grade 3 anaplastic astrocytoma progressed after radiotherapy (54 Gy) to an unresected left cerebral hemisphere tumour. Therapy with temozolomide was commenced 44 days after the completion of radiotherapy. A 30% reduction in the product of the

© Cancer Research Campaign 1998

A

C

B

**Figure 6** Response to therapy with SCH 52365 as imaged by CT scan. **A** was taken at relapse, **B** after two courses of SCH 52365 (partial response) and **C** at the time of maximal response (at 17 weeks), in a 9-year-old girl with a glioblastoma, which recurred 62 weeks after total excision and radiotherapy two maximal perpendicular diameters of the tumour was observed after two cycles of therapy, and no tumour was detectable after 52 weeks of therapy. During this time, the patient showed continued improvement in her right-sided hemiplegia, and therapy with dexamethasone was withdrawn by week 14. The patient relapsed with disease in the contralateral (non-irradiated) cerebral hemisphere 5 months after temozolomide was discontinued.

Another patient, a 9-year-old girl with a right cerebral giant-cell glioblastoma, had a total resection and radiotherapy (54 Gy) 14 months before her relapse. After two courses of therapy with temozolomide, a 70% reduction in the product of the two maximal perpendicular diameters of the tumour was observed, and the maximum response of a 81% reduction was observed after 16 weeks (Figure 6). This patient became asymptomatic during the course of her therapy with temozolomide, allowing the withdrawal of dexamethasone by week 8. The partial response lasted for 20 weeks. One further patient had stable disease lasting 4 months.

All patients with primitive neuroectodermal tumours (six), ependymoma (one), rhabdomyosarcoma (one), chondrosarcoma (one), osteogenic sarcoma (one), pancreatic neuroendocrine tumour (one) and haemangiopericytoma (one) progressed after one or two courses of therapy.

### DISCUSSION

This phase I study has determined the dose-limiting toxicities, MTD and pharmacokinetics of oral temozolomide in children who have not received prior therapy with CSI or nitrosoureas. Although the MTD of temozolomide given orally for 5 days has been established in adults (Newlands et al. 1992; Brada et al. 1995; Reidenberg et al.

© Cancer Research Campaign 1998

1996), it was necessary to define the MTD of temozolomide in children, as the MTD of anti-cancer agents can be markedly different between children and adults (Marsoni et al, 1985).

As previous studies in children have shown that the MTD of anti-cancer agents can be influenced by the intensity of prior therapy (Pearson et al, 1994), patients were initially stratified according to whether or not they had received prior CSI or nitrosourea therapy. However, entry to the prior CSI/nitrosourea arm of the study was closed after the initial two dose levels because of poor recruitment.

Because, at the beginning of this phase I study, SCH 52365 had not been fully evaluated in an adult phase I trial, the starting dose level for both arms of the trial was 50% of the adult MTD established with the original CRC formulation (Newlands et al, 1992). The finding of thrombocytopenia as the dose-limiting toxicity in this study is in keeping with the original adult phase I trial of oral temozolomide, in which myelosuppression was found to be dose limiting. As in the adult phase I study (Newlands et al, 1992), thrombocytopenia occurred 20 days after the beginning of the first treatment cycle and persisted for between 7 and 42 days. Although, in this present study, dose-limiting myelosuppression was not observed in cycle 1 in one patient at the 1200 mg $m^{-2}$ dose level, grade 4 thrombocytopenia was observed in this patient on subsequent courses, necessitating a dose reduction. Furthermore, a recent phase I study of SCH 52365 in adults has also established thrombocytopenia as the dose-limiting toxicity, with an MTD of 1000 mg $m^{-2}$ per cycle (Brada et al, 1995). The importance of prior therapy has also been reported, with a lower MTD reported for patients receiving heavy prior chemotherapy (Reidenberg et al, 1996; Dhodapkar et al, 1997). For this present study, for patients who had not received prior CSI or nitrosoureas, no relationship between the intensity of prior therapy and dose-limiting toxicity could be determined.

The other main toxicities associated with temozolomide in this paediatric phase I study were nausea and vomiting. These symptoms were usually limited to day 1 of the first cycle and were easily controlled with standard antiemetic therapy. The toxicities of headache observed in Arm A and Arm B, and ataxia, pain and constipation in Arm B were thought to be due to the patients' underlying CNS tumours, or to treatment for constipation. Oral temozolomide was very well tolerated, with all but one child able to swallow the capsules. However, the necessity of swallowing whole capsules meant that very young children could not be entered into the study. There were no deaths attributed to temozolomide-related toxicity.

The results of the pharmacokinetic analyses of this phase I study were compatible with the findings from the adult phase I studies (Newlands et al, 1992; Schering-Plough Research Institute, 1996), with rapid absorption, rapid elimination, no accumulation on day 5 and a linear increase in peak plasma concentration and systemic exposure with increasing dose. Overall, the plasma concentrations of temozolomide in paediatric patients were approximately 15–30% higher than those observed at similar dose levels in the adult phase I study of SCH 52365 (Brada et al, 1995; Schering-Plough Research Institute, 1996). Similarly, systemic exposure (AUC) to temozolomide was higher in paediatric patients, with an increase of approximately 40% compared with adult patients (Schering-Plough Research Institute, 1996). Moreover, an important characteristic of temozolomide is that the pharmacokinetics are reproducible in this patient population, as demonstrated by the small interpatient variability in systemic exposure. Other pharmacokinetic parameters in this paediatric study, i.e. $T_{max}$ and $t_{1/2}$, were similar to those observed in adults. As in the adult phase I study of SCH 52365, the urinary clearance of temozolomide was small compared with apparent total body clearance. However, urinary excretion may have been underestimated because of the breakdown of temozolomide in the urine, before micturition and subsequent stabilization of temozolomide by acidification of the urine.

In an adult phase II study of temozolomide in primary brain tumours (O'Reilly et al, 1993), major clinical improvement was found in six out of ten evaluable patients with high-grade astrocytomas who had relapsed after radiotherapy. This was accompanied by a marked improvement in radiological appearance in five out of ten patients. Moreover, reduction in the size of tumours was also seen in four out of seven patients with unirradiated astrocytomas (O'Reilly et al, 1993), confirming the suggestion of activity against HGA observed in the original phase I study (Newlands et al, 1992). Activity in this paediatric study has been demonstrated in patients with high-grade astrocytoma and brain stem glioma, with measurable and confirmed responses (CR or PR) found in two out of five cases of high-grade astrocytoma, and one out of ten cases of diffuse intrinsic brain stem glioma. Although temozolomide was commenced 24 days after the completion of radiotherapy for the responding patient with a brain stem glioma, it was felt that the clinical and radiological responses seen were attributable to therapy with this agent. Response assessment in patients with CNS tumours is known to be difficult; however, only neuroradiological responses were considered in this study. All tumour sizes were measured independently by the local and two central radiologists to ensure a non-biased conclusion. Maximal responses were seen after more than two cycles of therapy in these three patients, in whom the greatest response occurred at 16, 32 and 52 weeks. Two patients with stable disease after two courses subsequently achieved a CR and a PR.

The cytotoxicity of temozolomide is thought to relate primarily to methylation of guanine at the $O^6$ position, and this DNA lesion is repaired by the protein $O^6$-alkylguanine DNA alkyltransferase (Catapano et al, 1987). The finding that this repair protein cannot be detected in 22% of primary brain tumours (Citron et al, 1991) and that the levels of MGMT in human tumour cells correlate with temozolomide cytotoxicity (Wedge et al, 1996) may prove to be clinically important observations. Indeed, rapid and sustained depletion of MGMT occurs in adult patients with a 200 mg $m^{-2}$ bolus dose of temozolomide followed by a twice-daily oral regimen for 5 days (Gerson et al, 1996).

As the prognosis for both high-grade astrocytoma and brain stem glioma in children is poor with current therapy, phase II evaluation of temozolomide is warranted for both of these conditions. Consideration should also be given to evaluating activity of temozolomide in other poor-risk tumours, such as neuroblastoma, in which dacarbazine is known to be active.

### ACKNOWLEDGEMENTS

The authors gratefully acknowledge the participation of the following UKCCSG centres during this study: Royal Victoria Infirmary, Newcastle upon Tyne NE1 4LP, UK; Royal Marsden Hospital, Sutton, Surrey SM2 5PT, UK; St James University Hospital, Leeds LS9 7TF, UK; Alder Hey Childrens Hospital, Liverpool L12 2AP, UK; Birmingham Childrens Hospital, Birmingham B16 8ET, UK; Southampton General Hospital, Southampton SO9 4XY, UK; University Hospital, Queens Medical

© Cancer Research Campaign 1998

Centre, Nottingham NG7 2UH, UK; Christie Hospital NHS Trust, Manchester M20 4BX, UK; and the Bristol Royal Hospital for Sick Children, Bristol BS2 8BJ, UK.

### REFERENCES

Bleehen NM, Newlands ES, Lee SM, Thatcher N, Selby P, Calvert AH, Rustin GJS, Brampton M and Stevens MFG (1995) Cancer Research Campaign Phase II trial of temozolomide in metastatic melanoma. *J Clin Oncol* **13**: 910–913

Brada M, Moore S, Judson I, Batra VJ, Quartey P and Dugan M (1995) A Phase I study of SCH 52365 (temozolomide) in adult patients with advanced cancer. *Proc Am Soc Clin Oncol* **14**: 484

Catapano CV, Broggini M, Erba E, Ponti M, Mariani L, Citti L and D'Incalci M (1987) In vitro and in vivo methazolastone-induced DNA damage and repair in L-1210 leukaemia sensitive and resistant to chloroethylnitrosoureas. *Cancer Res* **47**: 4884–4889

Citron M, Decker R, Chen S, Schneider S, Graver M, Kleynerman L, Kahn LB and White A (1991). $O^6$-alkylguanine DNA methyltransferase in human normal and tumour tissue from brain, lung, and ovary. *Cancer Res* **51**: 4131–4134

CPMP Working Party on the efficacy of Medicinal Products (1990) European Community Commission notes for guidance: Good Clinical Practice for trials of Medicinal Products in the European Community. *J Pharmacol Toxicol* **67**: 361–372

Dhodapkar M, Rubin J, Reid JM, Burch PA, Pitot HC, Buckner JC, Ames MM and Suman VJ (1997) Phase I trial of temozolomide (NSC 362856) in patients with advanced cancer. *Clin Cancer Res* **3**: 1093–1100

Gerson SL, Spiro TP, Reidenberg P, Schupp J, Liu L, Haaga J, Majka S, Statkevich P, Batra V, Dugan M and Willson JKV (1996) Rapid depletion of $O^6$-alkylguanine DNA alkyltransferase with twice daily oral temozolomide (SCH 52365) in patients with advanced cancer. *Proc Am Soc Clin Oncol* **15**: 178

Gundersen S, Aamdal S and Fodstad O (1987) Mitozolomide (NSC35451), a new active drug in the treatment of malignant melanoma. Phase II trial in patients with advanced disease. *Br J Cancer* **55**: 1249–1250

Korn EL, Midthune D, Chen TT, Rubinstein LV, Christian MC and Simon RM (1994) A comparison of two Phase I trial designs. *Stat Med* **13**: 1799–1806

Liu L, Markovitch S and Gerson SL (1996). Mismatch repair mutations override alkyltransferase in conferring resistance to temozolomide but not to 1,3-Bis(2-chloroethyl)nitrosourea. *Cancer Res* **56**: 5375–5379

Marsoni S, Ungerleider R, Hurson S and Hammershaimb L (1985) Tolerance to antineoplastic agents in children and adults. *Cancer Treat Rep* **69**: 1263–1269

Mosteller RD (1987) Simplified calculation of body-surface area. *New Engl J Med* **317**: 1098

Newlands ES, Blackridge GRP, Slack JA, Rustin GJS, Smith DB, Stuart NSA, Quaterman CP, Hoffman R, Stevens MFG, Brampton MH and Gibson AC (1992). Phase I trial of temozolomide (CCRG 81045: M&B 39831: NSC 362856). *Br J Cancer* **65**: 287–291

O'Reilly SM, Newlands ES, Glaser MG, Brampton M, Rice-Edwards JM, Illingworth RD, Richards PG, Kennard C, Colquhoun IR, Lewis P and Stevens MFG (1993) Temozolomide: a new oral cytotoxic chemotherapeutic agent with promising activity against primary brain tumours. *Eur J Cancer* **29A**: 940–942

Pearson ADJ, Price L, Hofer C, Lashford L, Lewis IJ, Mott M, Newell DR, Pinkerton CR, Stevens R, Stevens R and Windebank K (1994) Phase I study of continuous intravenous infusion of low dose thiotepa in children. *Med Paediatr Oncol* **23**: 270

Reidenberg P, Villalona M, Eckhardt G, Rodriguez G, Burris H, Von Hoff D, Statkevich P, Batra V, Dugan M and Eckhardt J (1996) Phase I clinical and pharmacokinetic study of temozolomide in advanced cancer patients stratified by extent of prior therapy. *Proc 9th NCI/EORTC Symp New Drugs Cancer Ther* **7**: 344

Schering-Plough Research Institute, Department of Drug Metabolism and Pharmacokinetics (1996) A Phase I study of SCH 52365 in adult patients with advanced cancer. Appendix B-1: Pharmacokinetics of SCH 52365 (temozolomide). Schering-Plough Research Institute, Report Number 193-114-01.

Stevens MF, Hickman JA, Langdon SP, Chubb D, Vickers L, Stone R, Baig G, Goddard C, Gibson NW and Slack JA (1987) Antitumour activity and pharmacokinetics in mice of 8-carbamoyl-3-methyl-imidazo [5,1-d]-1,2,3,5-tetrazin-4(3H)-one (CCRG 81045; M&B 39831), a novel drug with potential as an alternative to dacarbazine. *Cancer Res* **47**: 5846–5852

Wedge SR, Porteus JK and Newlands ES (1996) 3-aminobenzamide and/or $O^6$-benylguanine evaluated as an adjuvant to temozolomide or BCNU treatment in cell lines of variable mismatch repair status and $O^6$-alkylguanine-DNA alkyltransferase activity. *Br J Cancer* **74**: 1030–1103

WHO (1979) *WHO Handbook for Reporting Results of Cancer Treatment*. WHO Offset Publication no. 48. World Health Organization: Geneva

Woll PJ, Crowther D, Johnson PWM, Soukop M, Harper PG, Harris M, Brampton MH and ES Newlands (1995) Phase II trial of temozolomide in low-grade non-Hodgkin's lymphoma. *Br J Cancer* **72**: 183–184

© Cancer Research Campaign 1998